# Principles and Perspectives of Radiographic Imaging with Muons

**DOI:** 10.3390/jimaging7120253

**Published:** 2021-11-26

**Authors:** Luigi Cimmino

**Affiliations:** 1 Department of Physics, University of Naples Federico II, 80126 Napoli, Italy; cimmino@na.infn.it; 2 Division of Naples, Italian National Institute for Nuclear Physics, 80126 Roma, Italy

**Keywords:** muography, radiographic imaging, three-dimensional reconstruction method, muon flux modelling

## Abstract

Radiographic imaging with muons, also called Muography, is based on the measurement of the absorption of muons, generated by the interaction of cosmic rays with the earth’s atmosphere, in matter. Muons are elementary particles with high penetrating power, a characteristic that makes them capable of crossing bodies of dimensions of the order of hundreds of meters. The interior of bodies the size of a pyramid or a volcano can be seen directly with the use of this technique, which can rely on highly segmented muon trackers. Since the muon flux is distributed in energy over a wide spectrum that depends on the direction of incidence, the main difference with radiography made with X-rays is in the source. The source of muons is not tunable, neither in energy nor in direction; to improve the signal-to-noise ratio, muography requires large instrumentation, long time data acquisition and high background rejection capacity. Here, we present the principles of the Muography, illustrating how radiographic images can be obtained, starting from the measurement of the attenuation of the muon flux through an object. It will then be discussed how recent technologies regarding artificial intelligence can give an impulse to this methodology in order to improve its results.

## 1. A Brief Overview

The muographic methodology is used for imaging the interior of a body, exploiting the high penetration capacity of cosmic muons [1,2,3,4,5,6]. Two different techniques are currently referred to as Muography and used to measure the density of the target object: absorption Muography and deviation Muography. Deviation Muography is usually used for the detection of high density materials in small size objects and is based on the multiple Coulomb scattering experienced by muons when passing through matter [7,8,9,10].

In absorption Muography, the attenuation of the muon flux is used to measure the absorption of muons into matter. The measured number of muons is used to obtain a two-dimensional scatter plot of the density of a target volume. Large volumes can be imaged with this technique as sensed by the pioneering works provided by George [11] in 1955, who obtained a measurement of the overburden of a tunnel, and by Alvarez [12], who first provided a muograph of the Chefren pyramid. The applications of muography in the volcanological field [13,14,15,16,17,18,19,20] are very frequent, and from the 1990s onwards they have contributed to the revival of the methodology. Recent works in absorption Muography concern the study of the pyramid of Cheops (Kufhu) [21] and other cultural heritages [22], the discovery of cavities inside a hill [23,24], the exploration of archaeological sites [25,26], and the imaging of tidewater glaciers [27] and of nuclear reactors [28,29,30]. Absorption Muography is nowadays used in highly disparate fields, with techniques that differ not only on the basis of the size and density of the objects, but also according to the setup of the detectors and the technologies used for their realization and performance optimization. Detectors normally used in the muographic field derive directly, or in any case, are adapted from those used for the detection of ionizing radiation in high energy physics. Detailed descriptions of the detection technologies used in muon radiography can be found in [1,2,3].

Natural muons are generated from the so-called primary cosmic radiation. The primary cosmic rays are mainly composed of stable charged particles and of nuclei with lifetimes of millions of years. Once this radiation reaches the Earth atmosphere, it interacts with the atoms all along the stratosphere, the part of Earth’s atmosphere that extends for 40 km from 10 km above sea level and below, producing showers of new particles called secondary cosmic rays. At sea level, the secondary charged radiation is mainly composed of electrons, positrons, muons and protons. Muons are the most abundant component of secondary radiation.

**Figure 1:**
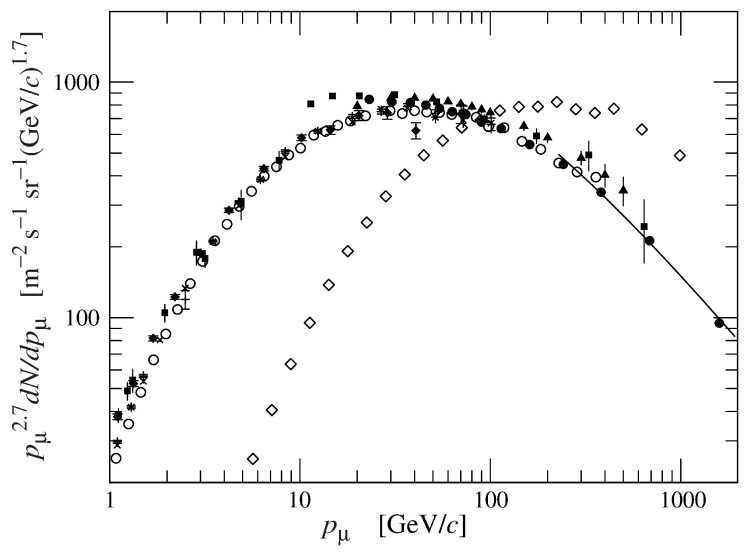
Energy spectra of muons at $0^{\circ }$ (black dots) and at $75^{\circ }$ (white dots) as in [31].

The decay of intermediate mesons π and *K* is such to generate high energy muons, which can reach several tens of TeV and more. They are leptons, ∼200 times heavier than the electrons, with a large energy spectrum and high penetrating power. The fraction of muons capable of crossing a few thousand meters of matter increases in the range of the angles close to the horizon. Figure 1 shows the fluxes of vertical and quasi-horizontal muons; it shows an upper shift in the energy of the flux spectrum at $75^{\circ }$ with respect to the flux at $0^{\circ }$, as a function of the muon momentum and, hence, of their energy. This means that the horizontal muon flux is more energetic than the vertical one. However, the latter has a higher average intensity than the former.

The typical rate of muons at sea level is 1 particle per minute per square centimeter area and the detection of muons on the Earth’s surface is due to the time dilatation effect of the theory of relativity, because their mean lifetime in the muon’s proper system of reference is τ=2.2 µs. Muons are mostly generated in the range of altitudes between 26 km and 15 km a.s.l. and the mean energy of the muons at sea level is 4 GeV, after they lose about 2 GeV reaching the ground. According to the theory of relativity, since the muon’s mass at rest is $m_{0}=105.7$ MeV/c${}^{2}$, for a muon produced with energy E=6 GeV, one obtains

$$\gamma =\frac{E}{m_{0}c^{2}}=\frac{6\;\mathrm{GeV}}{105.7\;\mathrm{MeV}}=56.8\;t=\gamma \times \tau =56.8\times 2.2\;\mu s=125.0\;\mu s$$

where γ is the relativistic term. Using the natural expression of γ(v), we obtain the muon speed v=0.9998c and thus the resulting decay length is approximately 37.5 km. This is reduced to about 25 km due to energy losses caused by the interaction with the atmosphere.

As for the interaction of muons with matter, we must refer to the probability for a muon not to interact over a distance *x*. This probability is given by $e^{-x/\lambda }$ [32], where the mean absorption length parameter $\lambda ={\left(\rho \;\sigma \right)}^{-1}$, depends on the density of the material and the cross section σ that is commonly represented as a function of the energy *E* of the incoming particle and its value represents the probability whereby a certain process occurs. In the case of muons, the thickness *x* crossed is much larger than the mean absorption length and the number of interactions of the muons with the matter is of order x/λ. So, to quantify the average energy loss per unit length experienced by muons, the stopping power −dE/dx is used.

A complete analysis concerning a muon’s interaction effects inside matter can be found in the work by Groom, Mohkov and Strikanov [33]. Muons with energy from 100 MeV up to 100 GeV are considered minimum ionization particles and the energy loss rate is constant at the first order [34]. So, the stopping power for muons with energy above some GeV is

$$-\frac{dE}{dx}\approx 2.1\;\mathrm{MeV}\;g^{-1}\;{\mathrm{cm}}^{2}$$

in dense materials, while it is less than 2 MeV g${}^{-1}$ cm${}^{2}$ in air, which explains the 2 GeV energy loss of the muon in reaching the surface of the Earth.

## 2. Modeling the Muon Flux

The flux Φ, in m${}^{-2}$ sr${}^{-1}$ s${}^{-1}$, of the primary cosmic rays depends on the energy *E* of the incoming particles, by means of the relationship $\Phi =P_{0}\;E^{-2.7}$ [35]. The factor $P_{0}$ turns out to be approximately $1.8\times 10^{4}$, with the units m${}^{-2}$ sr${}^{-1}$ s${}^{-1}$ GeV${}^{2.7}$, for nucleons with energy in the range of few GeV up to hundreds of TeV [36].

Starting from the primary radiation’s components, hadronic processes generate pions (π) and kaons (*K*), which weakly decay, generating muons. The main reactions producing muons involve charged pions, through decays

$$\begin{array}{c} \pi^{-}\to \mu^{-}\;\bar{\nu }\\ \pi^{+}\to \mu^{+}\;\nu \end{array}$$

with a ∼100% branching ratio, where a pion’s mean lifetime is 26.03 ns. The other direct source of muons are charged kaons, which produce them in a similar way, with the branching ratio 63.5% (mean lifetime is 12.38 ns).

Although the secondary cosmic radiation is not isotropic, we assume that the muons, since they were produced, have traveled in a straight line through the atmosphere and that the variation of the muon flux with the zenith angle is negligible, if detectors with small angular aperture are used. This hypothesis is not entirely true in general, but is a common practice in the field of Muography. Muon detectors used in Muography track the trajectory of muons without measuring their energy, so a 1 GeV muon is completely equivalent to any other muon of different energy. Furthermore, it is usually preferred to have low angular acceptance in order to better focus the target region, and therefore differences in the muon flux at different elevation angles are also considered to be completely negligible. Regardless of the setup, when pointing to the target region, a commonly measured acquisition rate is around 10 Hz per square meter of detector surface, with relative distance between the outermost tracking planes of the order of one meter. Neglecting the dependence of the flux by the azimuth angle φ and being the attenuation of the flux an exponential function of the depth *x* in the atmosphere with mean attenuation length λ, one has $i\left(\theta ,\varphi \right)=i_{⊥}\;e^{-x/(\lambda \;cos\theta )}$, where $i_{⊥}\left(\frac{x}{cos\theta }\right)$ is the vertical flux as a function of the slant depth $\frac{x}{cos\theta }$. Therefore, if the flux varies only as a function of *x*, the infinitesimal flux at depth *x* is considered to be the vertical flux with respect to the slant depth x/cosθ.

The number of cosmic muons crossing the unit area per unit of time is, by definition, the muon flux $\phi =\int_{0}^{2\pi }\int_{0}^{\pi /2}i\left(\theta ,\varphi \right)\;sin\theta \;d\theta \;d\varphi =\int i\left(\Omega \right)d\Omega$, where θ and φ are, respectively, the zenith angle and the azimuth, and i(θ,φ) is the infinitesimal flux per unit solid angle. The latter can be taken as a function of the thickness *x* passed by a cosmic ray at a given θ angle and, without loss of generality, we consider

(1) $$i_{\theta }\left(x\right)=i_{⊥}\left(\frac{x}{cos\theta }\right).$$

The Equation (1) represents a good approximation of the flux for $\theta >70^{\circ }$, while in the case of muon energies $E_{\mu }$ less than tens of GeV and for zenith angles smaller than $70^{\circ }$, experimental data [37] lead us to consider

(2) $$i_{\theta }\left(x\right)=i_{⊥}\;cos^{n}\theta$$

being $\bar{n}=1.85$ the mean value of *n* when we consider angles up to $70^{\circ }$ and muons with energy greater than 1 GeV, which allows us to take n=2 over a large energy range; $i_{⊥}=i_{\theta =0}\left(0\right)$ is the vertical muons flux and at sea level it is ∼70 m${}^{-2}$ s${}^{-1}$ sr${}^{-1}$ [37,38].

**Figure 2:**
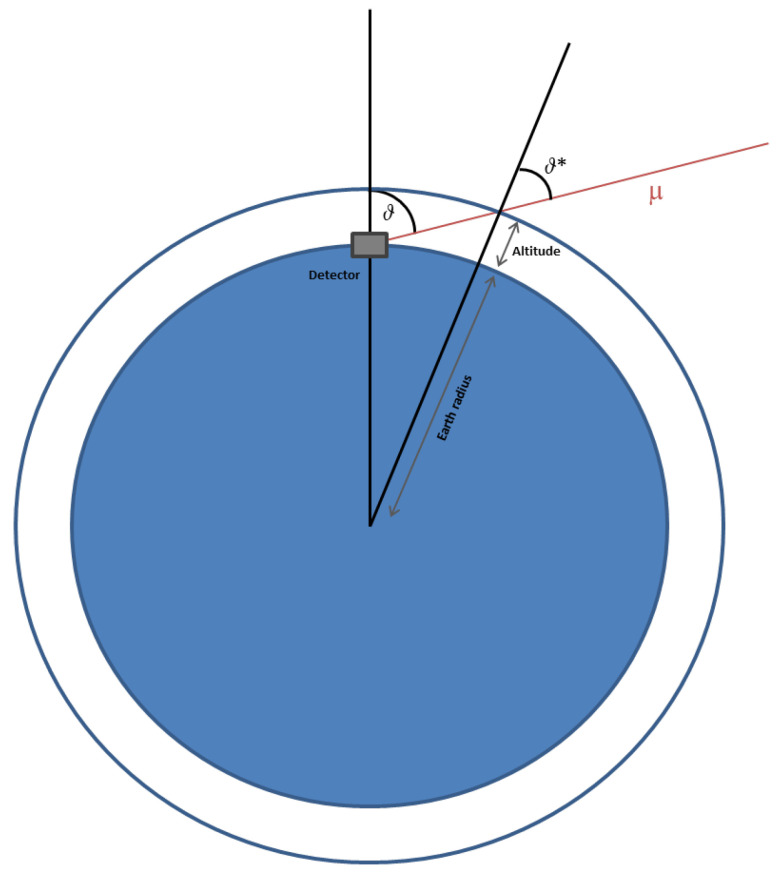
The angles θ and $\theta^{*}$ formed by an incoming muon, respectively, with the normal axis to the earth surface and with the normal axis to the top of the atmosphere.

The differential flux per unit area and time is $d\varphi =P_{\mu }E^{-2.7}dE_{\mu }d\Omega$, where the factor $P_{\mu }$ takes into account the contribution of the particles π and *K* to the muons flux and it results

$$P_{\mu }\propto {\left(1+\frac{1.1\;E_{\mu }\;cos\theta }{\epsilon_{\pi }}\right)}^{-1}+0.054\;{\left(1+\frac{1.1\;E_{\mu }\;cos\theta }{\epsilon_{\kappa }}\right)}^{-1}$$

where $\epsilon_{\pi }=115$ GeV and $\epsilon_{\kappa }=850$ GeV are energy values above which the two mesons respectively decay before interacting with the atmosphere. The same proportionality holds for muons arriving with $\theta >70^{\circ }$, but, looking at the Figure 2, we have to consider

(3) $$cos\theta^{*}=\sqrt{1-\frac{1-cos^{2}\theta }{{\left(1+r\right)}^{2}}}$$

which includes *r* as the dimensionless constant ratio between the muon’s production altitude *H* at zenith angles close to the horizon and the Earth radius *R*, both in the same unit [39]. Here θ and $\theta^{*}$ are such that 0≤cosθ≤1 and $0.103\leq cos\theta^{*}\leq 1$ [40].

There are different models, both analytical and theoretical, to describe the muon energy spectra [3,41,42,43]. The most basic theoretical model, which does not take into account the Earth’s curvature and the attenuation of the muon flux along the atmosphere, is commonly attributed to Gaisser [35,36], and expressed in terms of the Equation (3)

(4) $$\begin{array}{c} \frac{dN}{dE_{\mu }d\Omega }\approx 0.14\times 10^{4}E_{\mu }^{-2.7}\;[{\left(1+\frac{1.1\;E_{\mu }\;cos\theta }{\epsilon_{\pi }}\right)}^{-1}+\\ +0.054\;{\left(1+\frac{1.1\;E_{\mu }\;cos\theta }{\epsilon_{\kappa }}\right)}^{-1}] \end{array}$$

in units of flux, i.e., m${}^{-2}$ s${}^{-1}$ sr${}^{-1}$. The most general expression

$$\begin{array}{c} \frac{dN}{dE_{\mu }d\Omega }\approx 0.14\times 10^{4}E_{\mu }^{\gamma }\;[{\left(1+\frac{1.1\;E_{\mu }^{*}\;cos\theta }{\epsilon_{\pi }}\right)}^{-1}+\\ +0.054\;{\left(1+\frac{1.1\;E_{\mu }^{*}\;cos\theta }{\epsilon_{\kappa }}\right)}^{-1}+\;p]\;W\left(E,\theta^{*}\right) \end{array}$$

is derived from Equation (4), which reduces to the original for $W(E,\theta^{*})=1$, γ=2.7 and p=0. Here, these three factors are, respectively, the probability of muons to reach Earth’s surface (survival rate of muons), the power index, and the ratio of prompt muons to pions [40]. Moreover, in the Gaisser model it is assumed $E_{\mu }^{*}=E_{\mu }$, where $E_{\mu }^{*}$ represents the initial energy of the muons and $E_{\mu }$ is their energy at the surface. This correction is important in the energy range above 10 GeV to take into account the energy loss due to the muons passage through the matter.

Therefore, using Equation (2), with n=2, and Equation (3), when we consider the entire spectrum of muons at each angle θ, i.e., for energies $E_{\mu }>\left(100/cos\theta^{*}\right)$ GeV and $E_{\mu }\leq \left(100/cos\theta^{*}\right)$ GeV, it is necessary to choose the parameterization that best suits the experimental data.

The Tang’s corrections [40] to the Gaisser model

$$W\left(E,\theta^{*}\right)=1.1\;{\left(\frac{90\;\sqrt{cos\theta +0.001}}{1030}\right)}^{\frac{4.5}{E_{\mu }cos\theta^{*}}}$$

$$E_{\mu }^{*}=E_{\mu }+2.06\times 10^{-3}\;\left(\frac{950}{cos\theta^{*}}-90\right)$$

$$p=10^{-4}$$

in which we are neglecting the contribution of muons with energy below tens of GeV, extends the Gaisser model to almost all θ angles. The additional factor in the second equation gives an estimate of the mean energy loss of muons along their slant paths. As shown in [39], a different parameterization is used based on Kudryavtzev’s work

$$W\left(E,\theta^{*}\right)={\left(\frac{120\;\cos\theta^{*}}{1030}\right)}^{\frac{1.04}{{\bar{E}}_{\mu }\;cos\theta^{*}}}$$

$$E_{\mu }^{*}=E_{\mu }+2.06\times 10^{-3}\;\left(\frac{1030}{cos\theta^{*}}-120\right)$$

$${\bar{E}}_{\mu }=E_{\mu }+1.03\times 10^{-3}\;\left(\frac{1030}{cos\theta^{*}}-120\right)$$

$$p=10^{-4}.$$

Another model often used [39,44], is the Matsuno model [45]

$$\frac{dN}{dE_{\mu }d\Omega }\approx A\;{\left(E_{\mu }^{*}\right)}^{\gamma }\;\left[\frac{r_{\pi }^{\gamma -1}\;\epsilon_{\pi }\;sec\theta }{E_{\mu }^{*}+\epsilon_{\pi }\;sec\theta }+\left(\frac{K}{\pi }\right)\;b_{r}\;\frac{r_{\kappa }^{\gamma -1}\;\epsilon_{\kappa }\sec\theta }{E_{\mu }^{*}+\epsilon_{\kappa }\;sec\theta }\right]\;W\left(E_{\mu },\theta \right)$$

where $r_{\pi }=0.78$ and $r_{\kappa }=0.52$ are the fractions of energy transferred to the muon, respectively, in a pion or kaon decay, $\frac{K}{\pi }=0.36$ represents the excess of the production rate of pions over kaons, $b_{r}=0.635$ is the branching ratio of kaons, and the muon energy $E_{\mu }^{*}=E_{\mu }+\Delta E$ takes into account the energy ΔE lost by the muon along its path. Furthermore, the survival probability of muons crossing a thickness *L* is expressed by

$$W_{\mu }\left(E_{\mu },\theta \right)=e^{\frac{\sqrt{R^{2}\;cos^{2}\theta +2\;R\;H+H^{2}}-R\;cos\theta }{6.2\;E_{\mu }}}.$$

**Figure 3:**
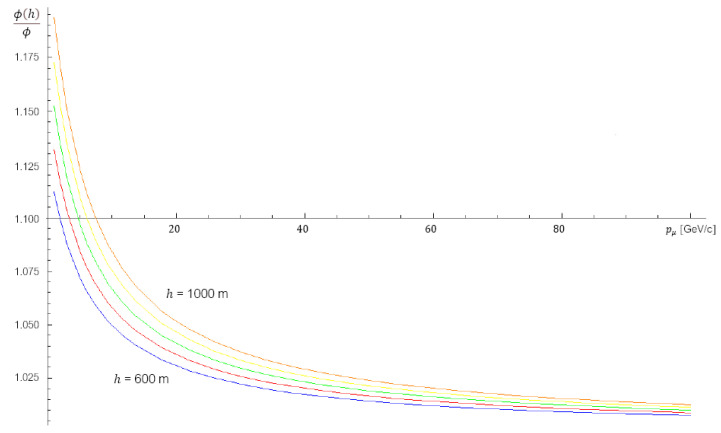
Trends in the ratio between the flux at different altitudes *h* and the flux at sea level as a function of the muons energy. The comparison of the curves gives the expected percentage change in the flux of muons at different altitudes.

A different choice of parameters can be made for this model too, although it should be noted that for *quasi-horizontal* flux the percentage variation among all models is of order ±10%. In addition to these models, we could take into account the effect of altitude within 1000 m. It results [46] that the muons flux at *h* meters above the sea level is

$$\Phi \left(h\right)=e^{\frac{h}{4900+750\;p_{\mu }}}\;\Phi$$

where $p_{\mu }$ is the momentum of the muon expressed in GeV/*c* and Φ is the flux of muons at the sea level described in terms of the corrected Gaisser’s model. This further correction takes into account an increase in the flux of muons with energies up to ∼100 GeV. As shown in Figure 3, the increment in muons flux at higher altitudes is of some percent in respect of the flux at the sea level and it is more appreciable at energy below 100 GeV.

## 3. The Technique of Radiographic Imaging with Muons

Muon radiography (Muography) is a technique based on the measurement of the absorption of cosmic muons in matter for the direct imaging of large structures. A muon detector looking at a target region detects the muons, tracks their routes and counts them. This allows the average density along the paths traveled by the muons to be calculated. In particular, vertical detectors are often required in some muographic applications due to the need to exploit the near horizontal muon flux, which is characterized by the high energy required to penetrate up to a few kilometers of matter.

In ideal conditions, the transmission as a function of the zenith and azimuth angles, is defined as

(5) $$T\left(\theta ,\varphi \right)=\frac{N(\theta ,\varphi )}{N_{exp}\left(\theta ,\varphi \right)}$$

that is the fraction between the number N(θ,φ) of muons that pass through the target and the number $N_{exp}\left(\theta ,\varphi \right)$ of expected ones without the target. The expected number of muons can be evaluated with Monte Carlo simulation or, alternatively, another procedure can be performed with a free sky data taking. The latter measurement has the advantage of including the detector efficiency factor, while in a Monte Carlo simulation it must be a priori well known. The measurement of the transmission in the free sky data acquisition involves two different measurements, in different time, so that the detector points to the target region and then, after it has been moved, to the open sky for the free sky data acquisition. The Equation (5) becomes the measured transmission

$$T_{m}\left(\theta ,\varphi \right)=\frac{\epsilon_{fs}}{\epsilon }\;\frac{t_{fs}}{t}\;\frac{N_{m}\left(\theta ,\varphi \right)}{N_{fs}\left(\theta ,\varphi \right)}$$

in which

$\epsilon_{fs}$ is the efficiency term of the detector in free sky data taking
ϵ is the efficiency term of the detector in the data taking
$t_{fs}$ is the time taken to perform the free sky data taking
*t* is the time taken to perform the data taking
$N_{m}\left(\theta ,\varphi \right)$ is the number of detected muons passed through the target
$N_{fs}\left(\theta ,\varphi \right)$ is the number of muons counted during the free sky run

Where the ratio $\frac{t}{t_{fs}}=1$, if *t* is normalized to the free sky data acquisition time. The efficiency terms can differ even if the detector used in the two different data taking is the same; in general, the efficiency term takes into account factors concerning the detector that affect both the acquisition and the analysis of the data.

On the other hand, we can calculate the expected transmission by means of the intensity $i(\theta ,\varphi ,E_{\mu })$ as a function of the muon energy $E_{\mu }$. By integrating this over all the energy spectrum we have

$$\phi =\int_{E}^{\infty }i\left(\theta ,\varphi ,E_{\mu }\right)\;dE_{\mu }$$

where *E* is, in general, a function of the angular coordinates and of the density of the material passed by the muon and represents the minimum energy that muons must have in order to be detected, therefore without being previously absorbed by the material through which they pass. We can compute the expected transmission as the ratio between the muon flux through a material of density and the muon flux in open sky, hence

$$T_{exp}\left(\theta ,\varphi \right)=\frac{\int_{E_{min}}^{\infty }i\left(\theta ,\varphi ,E_{\mu }\right)\;dE_{\mu }}{\int_{E_{0}}^{\infty }i\left(\theta ,\varphi ,E_{\mu }\right)\;dE_{\mu }}$$

with $E_{min}$ and $E_{0}$ being the minimum energy in the selected angular bin (θ,φ) of the target region and the open sky, respectively. A digital terrain model (DTM) of the target region is used to calculate the muon flux through it.

The relative transmission, whose scatter plot represents the muographic image, is given by

$$R\left(\theta ,\varphi \right)=\frac{T_{m}\left(\theta ,\varphi \right)}{T_{exp}\left(\theta ,\varphi \right)}$$

that reduces to the Equation (5) in the case in which the muon flux model is absolutely faithful to reality and normalized to the acquisition time, and the detector is faithfully reproduced with its efficiency in the process of calculating the expected flux.

Once the transmission map of the target has been measured, we can obtain the expected depth in kilometers water equivalent (kmwe), penetrated by muons at different zenith angles. Water constitutes an homogeneous medium, with unit density $\rho_{w}$ by default, and the depth measured using water can be easily compared to inhomogeneous material [34].

The expected depth in kmwe can be evaluated with a simulation in GEANT4 [47]. In this case, the expected transmission, as a function of the different path length at different zenith angles, is the ratio between the number of the survived muons and the number of injected muons. The energy spectrum at different θ of the injected flux is the Equation (5) and the curves, parameterized by the zenith angle passed in a given volume of material of known density $\rho_{m}$. If we assume the density of the water $\rho_{m}=1$, the real path length is equal to the depth in kilometers water equivalent penetrated by muons.

Once all values of the transmission in every angular bin have been measured, we obtain the related map of path length $x_{\theta }=\Lambda_{\theta }$. Since $x_{\theta }=L_{\theta }\;\rho$ and being $L_{\theta }$ the thickness of matter, of unknown density ρ, traversed by the muon at angle θ, we have

$$\rho =\frac{\Lambda_{\theta }}{L_{\theta }}$$

where $L_{\theta }$ is taken as in the DTM.

## 4. The 3D Reconstruction Technique and Expected Performance

By combining multiple radiographic images taken from different points of view, as successfully demonstrated in [48], it is possible to reconstruct an object seen under different angles. The main limit of the technique concerns the availability of places where detectors can be installed. Sometimes it may be impossible to have a large number of points of view, and above all it may not be possible to surround the target region. In addition, it must be borne in mind that due to the origin of the muon flux, the target region must always be at a higher height than that of the detector.

The reconstruction of a body with different density inserted in the target region takes radiographic images from each point of view, and identifies the signals belonging to each of them, which are in angular correspondence with each other. So, defining a grid of points in a cubic volume that encloses the region where the density discontinuity is found, according to the angular ranges, a point was considered to be located inside a discontinuity region if in each of the three radiographic images it corresponded to a direction lying inside a signal cluster.

**Figure 4:**
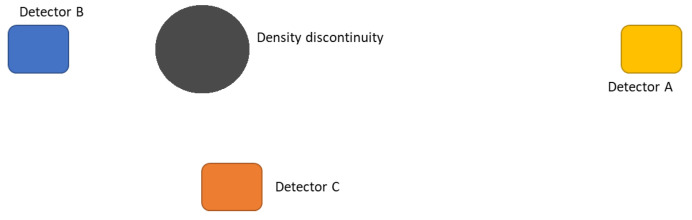
Schematic of the simulated scenario. Detectors (not in scale) placed in the positions A, B and C.

**Figure 5:**
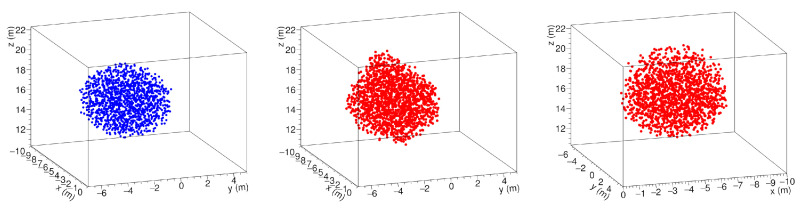
A spherical cavity (**left**) and its 3D reconstruction as seen from the observation point of the detectors C (**center**) and B (**right**) as in [48].

The procedure was tested by simulating the presence of a spherical cavity with 6 m diameter, seen by three points of view as in Figure 4. The result is shown in Figure 5 as seen by the detector C. The blue dots represent an ensemble of points distributed on the grid and located inside the cavity. The points satisfying the intersection criterion are denoted by red dots.

As it can be seen in Figure 5, the reconstructed cavity is deformed by the stereographic projection. This overestimates the size of the discontinuity, which appears to be larger in those directions in which there are no points of view. The origin of the observed halo is due to the fact that the criterion is satisfied also by points that, in a projection, appear in the shadow of the cavity, rather than inside. It is evident that the onset of this deformation in the reconstruction can be reduced, but not eliminated, by increasing the number of observation points. In any case, we must bear in mind that it is not possible to observe the target region from heights above the discontinuity zone.

## 5. Future Development

Machine learning is applied in every area of daily life, resulting in enormous advantages in terms of the quality of results and the speed with which they are obtained. One of the most impressive recent applicatiosn is the manipulation of low quality images in order to improve their quality and increase their resolution [49]. Even in medical imaging, machine learning is increasingly being used to achieve better diagnostic performance [50,51,52,53]. To go beyond the limitations of the 3D reconstruction technique, the use of machine learning could help to remove the stereographic halo from reconstructed images.

The precision with which the object measured in different radiographs is reconstructed in three dimensions, depends on the number of observation points and their location with respect to the measured object. In muographic applications, it is not always possible to choose the angles to solve ambiguities in the reconstruction, because of the limitations of the site. Moreover, placing the detector on a plane that is below the observed region would result in an enlargement of the top part of the object. The halo could be reduced by placing trackers at more favorable angles for triangulation, and by increasing the number of points of view beyond the minimum number of three used in this work.

Supervised machine learning techniques can help to overcome the limits of traditional 3D reconstruction. By simulating many shapes and training an ad hoc algorithm with these data, it is possible to predict and so reconstruct the shape of the measured object with a few radiographs taken from different angles.

More specifically, it is possible to design a supervised 3D reconstruction algorithm to train a machine learning model capable of predicting the ideal shape of an object starting from the features extrapolated from multiple radiographs. The synthetic data used in the machine learning procedure are produced through a set of simulations in which the features of the simulated objects vary to consider the most disparate cases. Furthermore, different datasets can be created taking into account a multitude of possible scenarios regarding the positioning of the detectors, the presence of other objects with different densities nearby, the exposure time, and so on. In the final stage, the radiographic images serve as input for the model, to predict the ideal shape of the object viewed from few different points of view. The real shape is obtained from the one reconstructed in 3D, which is cleaned up using the ideal shape predicted by the machine learning model.

**Figure 6:**
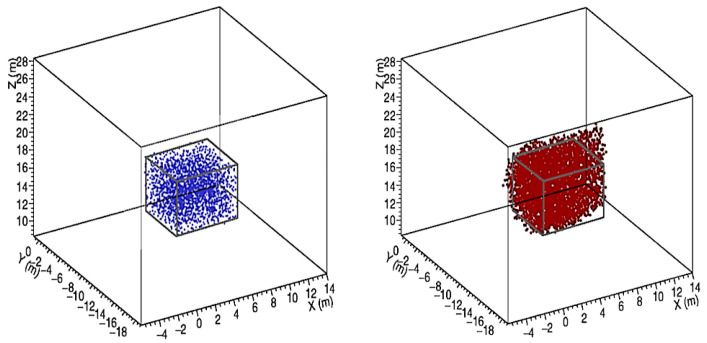
The cube (in gray) predicted by the supervised algorithm compared with the unknown object (left in blue) and with the 3D reconstruction of the unknown object (right in red).

Therefore, the simplest problem we are referring to is to determine the shape and size of the object identified in the target region by having *n* muographs that see the object from different angles $\alpha^{\left(j\right)}$, where j=1,⋯,n, and to have it reconstructed by means of 3D Muography. Given a certain arrangement of *n* detectors, *N* simple geometrical objects of different shapes and sizes are simulated, obtaining the dataset $\left(m_{i}^{\left(j\right)};x_{i}\right)$, where i=1,⋯,N. Using the same arrangement of detectors as in Figure 4, so that n=3, we produce the set of muographs $\left\{m^{\left(1\right)},m^{\left(2\right)},m^{\left(3\right)}\right\}$ obtained by a cloud of points randomly distributed in a cubic volume, which represents the unknown object *x*. The algorithm will predict that the shape and size of the muographed object are compatible with a 6 m side cube *X*, as in Figure 6. On the other hand, the same set of muographs is used to 3D reconstruct the unknown object *r*.

**Figure 7:**
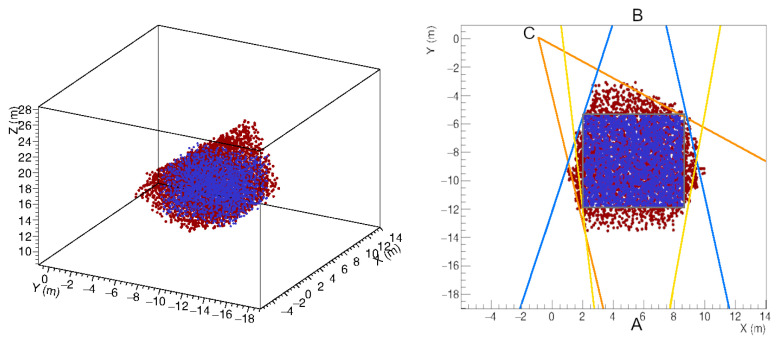
Denoising process of the 3D reconstructed object. The XY-view on the right shows the predicted object (blue), the 3D reconstructed object (red) and the reference object (gray) in the space constrained by the viewing angle of the detectors placed in positions A, B and C.

In this schema, the predicted cube *X* is the reference object, and a dataset $\left(R_{k};y_{k}\right)$ is created by randomly generating *M* distributions of points $R_{k}$, with k=1,⋯,M, constrained to the angles $\alpha^{\left(j\right)}$, as in a one-to-many conditional mapping from the single *X* to the set $\left\{R_{k}\right\}$. The objects $R_{k}$ are noisy representations of the ideal object *X* and the learning model is trained to reverse the $R_{j}$ in the reference object *X*. Thus, by giving the 3D reconstruction of the unknown object as input, the model will predict the cloud of points, compatible with the object *X*, which most closely approximates the observed object. Figure 7 shows the outcome of the denoising process operated on the 3D reconstructed object.

## 6. Conclusions

We described the technique of absorption Muography, providing all the steps that are necessary to have radiographic images of objects penetrated by muons. The modeling of the muon flux is crucial both for what concerns the feasibility study of the applications and for the obtaining of the radiographic images. The radiographic imaging with muons is as important as X-ray radiography. The direct imaging of the interior of geological buildings allows a detailed modeling which, at least in principle, would allow possible evolutions to be determined and, in some cases, could help prevent catastrophes.

Furthermore, the development of three-dimensional reconstruction techniques, which combine muographies of the same target region from different angles, constitutes one of the major advances in this research field. 3D muography not only allows one to determine the presence of structures with different densities, but also allows the reconstruction of their shape and the determination their size. This technique lends itself to any type of radiographic imaging, potentially constituting an alternative tool to standard techniques. If powered by artificial intelligence algorithms, it could in the future reach high levels of precision, starting from a small and partial number of observation points. 

## Data Availability

Not applicable.
